# The mediating role of controllability appraisals and coping strategies on adaptive functioning after job loss: a path model

**DOI:** 10.1038/s41598-022-19186-5

**Published:** 2022-09-01

**Authors:** Angela Socastro, Alba Contreras, Vanesa Peinado, Almudena Trucharte, Carmen Valiente, Carmelo Vazquez, Alvaro Sanchez-Lopez

**Affiliations:** Department of Personality, Evaluation and Clinical Psychology, Faculty of Psychology, Complutense University of Madrid, Somosaguas Campus, 28223 Madrid, Spain

**Keywords:** Health care, Occupational health, Psychology, Human behaviour

## Abstract

Job loss is a stressful event that increases the risk of experiencing depression and anxiety, especially during the initial months of unemployment. This study examined differences in psychological symptoms and resilient functioning accounted by employment status. The results pointed out that recently unemployed compared to currently employed individuals had lower levels of perceived controllability and resilience as well as higher levels of depression and anxiety. Path analyses showed that lower controllability appraisals at wave 1 of recently unemployed compared to employed individuals, in turn, predicted a lower use of active coping and reappraisal at wave 2, with the latter further accounting for lower levels in resilience. Higher use of distraction further mediated the relation between employment status and higher levels of depression and anxiety symptoms. Our findings demonstrate the importance of controllability appraisals and coping strategies used to promote adaptive psychological functioning following job loss.

## Introduction

Unemployment is one of the most crucial current social challenges^1^ having not only large socioeconomic impacts but also important consequences for citizens’ mental health^2^. Psychological disorders such as depression and anxiety are highly prevalent in the general population and are largely associated with the occurrence of adverse events or difficulties in living conditions such as unemployment^3^. Not surprisingly, job loss is seen as a major stressor that is consistently related to increased rates of depression, anxiety, and even suicidal behaviours (e.g.,^4^). Furthermore, massive layoffs and new scenarios of socioeconomic uncertainty have emerged during the last years because of the COVID-19 pandemic, and unemployment is forecasted to continue being a global societal problem into the next decade (e.g.,^1^). Altogether, this shows the importance of developing advanced frameworks to better understand the mechanisms that are implicated in resilience and adaptation to the psychological impact of unemployment. Only in this way new guidelines can be developed to promote psychological well-being in the face of job loss in scenarios of socioeconomic change. Therefore, the present longitudinal study aimed to a) test the impact of employment status (i.e., recent unemployment vs. current employment in individual levels of psychological symptoms and resilience during the initial months following job loss, and b) establish an empirically informed model of the psychological mechanisms that explain individuals' differences in the risk to develop psychological symptoms and impaired resilience to stress during initial months of unemployment.

### The psychological impact of unemployment across initial stages following job loss

Overall, meta-analytic work has consistently shown that unemployment is directly related to increases in depression, anxiety, and stress levels (e.g.,^5,6^). Such impacts tend to occur particularly during the initial months of unemployment following job loss, with some initial studies showing that 55% of psychological disorders occurring after job loss tend to emerge during the first 3 months of unemployment (e.g.,^7,8^). More contemporary research has also corroborated this evidence, showing that employment status (i.e., recently unemployed vs. employed) is the main predictor of psychopathological problems following job loss^9^. Individuals who lose their jobs report higher levels of both depressive and anxiety symptoms during the initial months of unemployment, in comparison to currently employed, as well as in comparison to re-employed people^10^. Specifically, depression levels start raising at 2 weeks or less following job loss and continue progressively increasing over time^11,12^, whereas the levels of anxiety and stress also reach peak levels within the first 3 months of unemployment^13,14^. Beyond specific forms of emotional symptomatology, global psychological distress indicators are also affected due to job loss, with several studies further showing that recently unemployed people experience impaired stress adjustment compared with both employed^15^ and reemployed people^16^. Ultimately, these psychological impacts during initial unemployment periods have long term consequences, as it has been shown that poor mental health levels following job loss predict larger periods of unemployment (e.g.,^17^).

### Mechanisms of mental health and stress adjustment during unemployment

The relevance of understanding which psychological mechanisms account for individual differences in mental health and stress adjustment during the initial stages of unemployment is thus of clear interest. In this line, processes such as controllability appraisals and the use of specific coping strategies have been proposed as central mechanisms of stress adaptation^18,19^. These processes have consequently been framed into models of coping with job loss, guiding research during the last decades (i.e.,^20^). Waters^20^ formulated an integrative model including cognitive appraisals, and coping and emotion regulation processes to improve psychological health and probabilities of reemployment following job loss. This framework states that differences in the cognitive appraisals about the unemployment situation and its controllability (i.e., individual’s capacities and resources to cope with it) can influence the type of coping and emotion regulation efforts that are carried out to deal with unemployment and its consequences. Ultimately, the differential use of such strategies would have a role in accounting for individual differences in the levels of psychological functioning of unemployed people and their opportunities for reemployment^20^.

Previous empirical studies considering individual differences in appraisals of perceived control in recently unemployed have found that higher controllability appraisals explained over a third of the variance in individuals’ psychological health levels and were positively associated with proactive job searches^21^. Klonowicz^22^ also found that an external locus of control in recently unemployed individuals-less than 2 months of unemployment- was positively related to negative affect and negatively related to life satisfaction levels. Furthermore, Creed et al.^23^ found that perceived control accounted for 50.6% of the variance in psychological distress of recently unemployed individuals and that this relation was consistent across longer periods of unemployment.

In line with the proposal of Waters^20^, other studies examining the emotional impact of unemployment have considered the role of individual differences in coping styles and the use of coping strategies following job loss. Initial studies in this matter showed that the use of problem-focused coping strategies was related to higher psychological distress at the beginning of the unemployment situation^24^. However, the use of problem-focused coping also predicted a higher probability of reemployment 3 months later^24,25^, highlighting its benefits in the mid-term. Other studies have considered other coping strategies, such as reappraisal (i.e., viewing stressful events from a different, more benign or positive, perspective^26^, or distraction (i.e., changing the focus of attention away from the stress-eliciting stimulus or undesirable internal states^27^. Specifically, Smari et al.^28^ found that the use of reappraisal was related to decreases in depression and anxiety, whereas the use of distraction as a form of avoidant coping was related to increases in these symptoms in recently unemployed.

### The interplay among controllability appraisals and coping strategies’ use to account for adaptive psychological functioning: general preliminary evidence

The above-mentioned research highlights the importance of studying both controllability appraisals and coping strategies’ use to understand the psychological impacts of job loss and unemployment and how to prevent them. Nevertheless, the influence of these variables in mental health after job loss has been studied in isolation, as independent predictors of psychological functioning in unemployed individuals. Interestingly, current frameworks, regarding stress regulation either in general^18^ or specifically in the context of job loss^20^, point out that these psychological processes are not independent, but potentially interrelated. More specifically, controllability appraisals might influence the way that different forms of coping and emotion regulation are enacted, ultimately accounting for individual differences in the development of psychological problems and stress maladjustment^18,20^.

Laboratory research suggests that reappraisal may be particularly effective to regulate the impact of stressors perceived as uncontrollable^29^. In contrast, in situations perceived as controllable, the use of problem-focused active coping has been identified as a potentially more effective strategy^30^. Further studies have also been conducted using methods of ecological evaluation of momentary contextual appraisals, analysing the influence of controllability appraisals in the use of coping strategies and resulting moods, as they occur during daily-life functioning. Results from this research show that perceived stress controllability are related to higher use of problem-solving and lower use of avoidant coping strategies^31^. Further, higher use of reappraisal specifically in situations perceived as uncontrollable has been found to account for lower depression and anxiety levels^32^.

### The current study

Based on preliminary evidence, the present study aimed to test how controllability appraisals and the use of different coping strategies (i.e., active coping, distraction, reappraisal) may act as intervening mechanisms to account for differential levels of psychological health (i.e., depression, anxiety) and stress adjustment (i.e., resilience) as a function of employment status (i.e., recently unemployed vs. employed). A longitudinal study was conducted, in which a large representative sample of the Spanish population was assessed two times across 30 days. Importantly, the study was conducted in the context of the beginning of the COVID-19 pandemic, a period that comprised high rates of layoffs and economic uncertainty. According to the Spanish Ministry of Work^33^, there were 26,573 layoffs and 347,858 employees were re-assigned to temporary workforce reduction programs (ERTEs) in Spain during those 30 days. Thus, the pandemic was a unique opportunity to test how job loss and resulting initial unemployment periods impacted subsequent psychological functioning, and how initial controllability appraisals and coping strategies following job loss ultimately might account for subsequent psychological health and stress adjustment.

Considering the revised literature, in this study, it was hypothesized that recently unemployed individuals would have higher levels of psychological symptoms (i.e., depression, anxiety) and lower levels of stress resilience compared to employed individuals (^9,15^; *Hypothesis 1*). Second, we expected that recently unemployed individuals with higher levels of psychological symptoms would have lower levels of perceived controllability (^22^; *Hypothesis 2*). As for the relations between controllability appraisals and the use of coping strategies, it was hypothesized that lower levels of controllability would be related to higher use of distraction and reappraisal and lower use of active coping strategies (^29–31^; *Hypothesis 3*). In terms of the relations between the use of coping strategies and psychological functioning outcomes, it was hypothesized that higher use of reappraisal would predict subsequent lower levels of depression and anxiety, and higher levels of resilience, whereas higher use of active coping and distraction would have the opposite relations (^18,32,34^; *Hypothesis 4*). Finally, in line with Waters^20^, an integrative *path model* of indirect effects was hypothesized (*Hypothesis 5*) where employment status was related to poor psychological functioning (i.e., higher depression and anxiety, lower resilience) indirectly through the influence of controllability appraisals and differential uses of coping strategies (i.e., employment status → controllability appraisals at Time 1 → coping strategies used from Time 1 to Time 2 → psychological functioning at Time 2). Thus, we hypothesized that recently unemployed people would have lower controllability appraisals, in turn using more reappraisal and distraction, and less active coping, in turn predicting differential levels of psychological functioning (i.e., depression, anxiety and resilience levels), in the hypothesized specific directions for each type of strategy.

## Method

### Participants and procedure

Our study used data gathered in a research project which analysed, as part of an international consortium, the psychological impact of COVID-19 in the Spanish population. The project was conducted online via Qualtrics software. Data used in this study comprised two surveys: T1, conducted from the 7th to the 14th of April 2020 and T2, conducted from the 7th to the 11th of May 2020.

The recruitment of the sample was carried out by a company that provides online samples for market research, using quota stratified sampling. Individuals between 18 and 75 years old were initially selected by sex, age and region quotas according to the National Institute of Statistics census of 1 January 2019. The original sample consisted of 1951 people of whom 1628 completed the two waves of the study. Of these 1628 subjects, 1376 persons were active working population in the first wave, the rest were retired, students or disabled people. To obtain the final database for the current study, focusing on differences via employment status, different filters were carried out. The filtering criteria were the following: (a) only participants ranging from 25 to 55 years were selected for the current study (this selection was established on the basis that this is the main age range in which unemployment rates tend to occur in the Spanish population;^35–37^); (b) participants whose employment status was ‘employed’ in the first wave and remained as ‘employed’ in the second wave were defined as the comparison group of ‘employed’ people; (c) finally, those individuals who lose their job during the follow-up (i.e., they indicated that they had lost their job due to the COVID-19 pandemic, and their employment status was ‘unemployed’ at both waves) were assigned to the group ‘recently unemployed, less than 3 months since job loss’, whereas individuals who were reemployed during follow-up (i.e., ‘unemployed’ at wave 1, and ‘employed’ at wave 2) were excluded from the final subsample.

Of the sample of 1376 individuals, a subsample of 668 individuals met the inclusion criteria. The final sample was comprised of 553 employed and 115 recently unemployed (i.e., less than 3 months since job loss) in the second wave. The mean age of the sample was 42.2 years and 41.2% were females.

### Materials

#### Depression levels

The Patient Health Questionnaire-9 (PHQ-9;^38^) was used to assess depressive symptom levels at both waves. It is a 9-item scale assessing depressive symptoms over the last two weeks, rated on a 4-point Likert scale, from 0 (not at all/less than 1 day) to 3 (nearly every day for 2 weeks). The overall score is the sum of items’ scores. Descriptive of this and the rest of questionnaires at each wave and their reliability indices are depicted in Table 1. In all cases, reliability indices for all measures at each wave were high.

**Table 1:** Cronbach’s alpha and test–retest reliability coefficients and descriptive statistics of the outcome measures at waves T1 and T2 (N = 668).

Cronbach’ α			Test–retest	T1				T2			
Measures	T1	T2		Mean	SD	Min	Max	Mean	SD	Min	Max
PHQ-9	.89	.90	.87	6.17	5.48	0	25	6.45	5.51	0	27
GAD-7	.93	.93	.87	5.76	5.21	0	21	5.43	5.06	0	21
BRS	.85	.86	.87	3,47	.76	1	5	3.49	.74	1	5

#### Anxiety levels

The Generalized Anxiety Disorder Scale (GAD-7;^39^) was used to assess anxiety levels in both waves. This is a 7-item scale assessing generalized anxiety levels in the last week, and it is rated on a 4-point Likert scale, from 0 (not at all) to 3 (nearly every day). The total score is obtained by the sum of scores from all the items.

#### Resilience levels

The Brief Resilience Scale (BRS,^40^) is a 6-item self-report scale that measures the perceived level of resilience in the face of stress and adversity through a 5-point Likert scale rated from 1 (strongly disagree) to 5 (strongly agree). The total score is the mean of the scores for the six items.

#### Controllability appraisal levels

An item of the Pemberton Happiness Index (PHI,^41^) was included to evaluate individual differences in controllability appraisals (i.e., perceiving that one has the resources or abilities to cope with ongoing problems). The item was: "I feel capable of solving most of my day-to-day problems". The item was rated on an 11- point Likert scale ranging from 0 (totally disagree) to 10 (totally agree). The score of this item was included in the study as a measure of controllability appraisal at wave 1.

#### Coping strategies’ frequency of use

The Coping Strategies Inventory (COPE,^42^) was included in wave 2, to index the degree of use of hypothesized coping strategies (i.e., distraction, reappraisal, active coping) to cope with stressors experienced during the period between wave 1 and wave 2 (i.e., 2 months). The original version of this multidimensional coping inventory comprises 28 items to assess the different ways in which people cope with stress, reporting responses on a 4-point Likert scale ranging from 1 (never) to 4 (almost all the time). In this project, the short version of the inventory was used, comprising 14 items focused on specific forms of coping. From that pool of 14 items, items comprising assessments of the coping strategies of interest, hypothesized as mediators in the full predictive path model (i.e., distraction, reappraisal, active coping) were considered for the current study. Based on the definitions of each strategy and previous exploratory factor analysis^43^ we selected specific items for active coping, reappraisal and distraction. The item for active coping was: "I have been focusing my efforts on doing things to improve the situation I’m in”. Two items were summed up to index reappraisal: "I have been trying to see it with different eyes, to make it seem more positive"; "I have been looking for something good in what's happening". These two items were positive correlated (*r* = 0.426, *p* < 0.001). Finally, two items were summed up to index distraction: "I have been resorting to working or other activities to get things out of my mind"; "I have been doing some things to think less about it, such as watching movies/series or TV”. These to items were also positive correlated (*r* = 0.356, *p* < 0.001).

### Statistical analyses

Data were analysed using the SPSS v.22 (IBM Corp, 2013). Analyses of differences between employed and unemployed people were first conducted for the variables of controllability appraisal, coping strategies, depression, anxiety, and resilience. For variables measured at both waves, differences due to time were also modelled. For this, a series of 2 (employment status: employed, recently unemployed) × 2 (time: wave 1, wave 2) repeated measures ANCOVAs were performed for controllability appraisal, depression, anxiety and resilience variables. The within-subject factor was Time (T1, T2), and the between-subject factor was Employment status (i.e., Employed, Recently Unemployed).

A series of Pearson’s bivariate correlations and chi-square analyses between employment status, controllability appraisal, coping strategies, depression, anxiety, and resilience variables were then performed to analyse the relations between them, depending on the nature of the variables (i.e., continuous or categorical). Based on relations supported in those initial analyses, we first tested a main path model, using a structural equation that included the full set of variables that were significantly correlated, in line with the paths of influence formulated in the hypotheses. In that model, employment status acted as exogenous variable, predicting future levels of depression, anxiety and resilience at wave 2 directly, but also indirectly through the influence of employment status on stress controllability (i.e., mediator 1) and the influence of the latter on individual differences in the use of active coping, reappraisal and distraction (i.e., mediators 2). Finally, in a second step, we tested a final path model where the same hypothesized set of paths were analysed after controlling for wave 1 levels of depression, anxiety, and resilience into the equation, allowing to further test paths of change from Time 1 to Time 2 in the outcome measures.

The estimation of standardized parameters of the paths models followed the full information maximum likelihood (FIML) estimation method. Adjustment of our models we tested using standard criteria^44^: (a) χ^2^: non-significant value; (b) χ^2^/gl: values lower than 2; (c) CFI and TLI: values ≥ 0.95; (d) RMSEA: values ≤ 0.05. Hypothesized mediation pathways (i.e., employment status → controllability appraisal → coping strategies → depression, anxiety and resilience), were tested via estimation of indirect effects within the full paths models. Structural equation models and resulting paths analyses were conducted using AMOS v18.0 (SPSS).

### Ethical approval

Ethical approval for the study was obtained from the Faculty Deontological Commission and was conducted in compliance with the Declaration of Helsinki. The reference of the approval was Ref. 2019/20-034. This approval contemplates obtaining an informed consent from each one of the participants, prior written and understandable information about the objectives and the procedure to which they are going to submit, the voluntary nature of their participation and their right to withdraw from the study at any stage of the study if they so wish. The investigator principal has also expressly agreed to respect the confidentiality of the information obtained and to safeguard it in accordance with current legislation.

## Results

Descriptive data of employment status and psychological measures of participants in the study are shown in Table 2.

**Table 2:** Descriptive data of the variables assessed in the study.

	Total sample (N = 668)	Employed (N = 553)	Recently unemployed (N = 115)
**Employment Status n (%)**			
Employed	553 (82.80)		
Unemployed	115 (17.20)		
Controllability wave 1, mean (SD)	7.63 (1.80)	7.78 (1.67)	6.90 (2.18)
Depression wave 1, mean (SD)	6.17 (5.48)	5.65 (5.10)	9.88 (6.63)
Anxiety wave 1, mean (SD)	5.76 (5.20)	5.38 (4.98)	7.59 (5.87)
Resilience wave 1, mean (SD)	3.47 (.76)	3.52 (.73)	3.21 (.83)
Controllability wave 2 levels, mean (SD)	7.55 (1.84)	7.72 (1.68)	6.72 (2.28)
Depression wave 2 levels, mean (SD)	6.45 (5.51)	5.74 (4.96)	9.88 (6.63)
Anxiety wave 2 levels, mean (SD)	5.43 (5.05)	4.81 (4.62)	8.43 (5.92)
Resilience wave 2 levels, mean (SD)	3.49 (.74)	3.55 (.73)	3.20 (.72)
Active Coping, mean (SD)	1.79 (.87)	1.77 (.86)	1.89 (.89)
Reappraisal, mean (SD)	3.30 (1.51)	3.26 (1.51)	3.50 (1.49)
Distraction, mean (SD)	3.50 (1.49)	3.43 (1.48)	3.85 (2.28)

### Differences due to employment status in each wave and across them

There were significant differences as a function of employment status in the second wave for distraction *t*(666) = -2.7; *p* < 0.05, with higher use of distraction in unemployed compared to employed participants. However, no main differences were found in the frequency of use of active coping, *t*(666) = -1.34, *p* = 0.178, and reappraisal as a function of employment status, *t*(166.46) = -1.54; *p* = 0.125.

**Figure 1:**
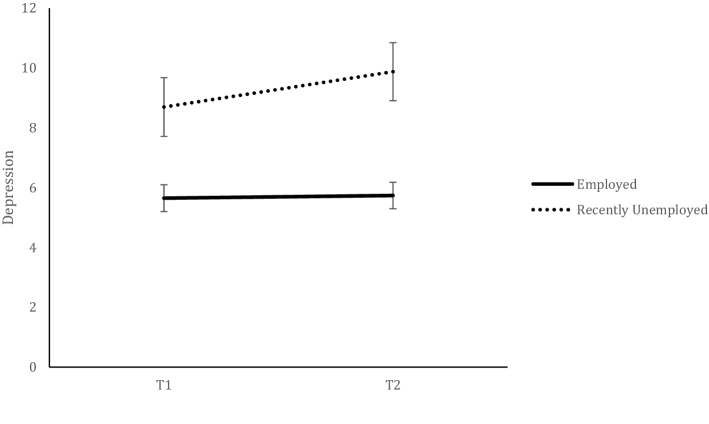
Depression levels of each employment category over time.

**Figure 2:**
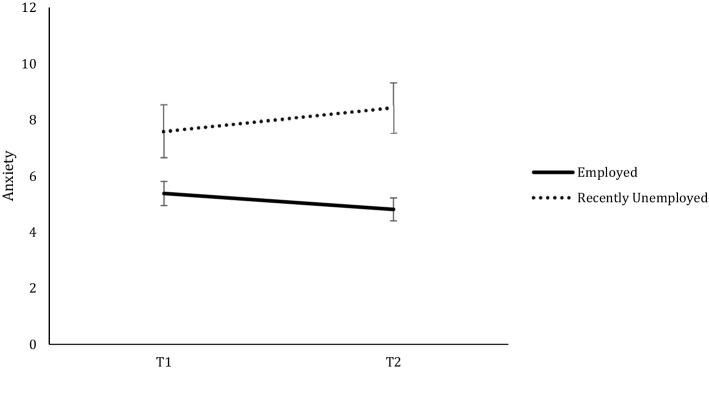
Anxiety levels of each employment category over time.

The results also showed significant differences between groups in depression. A significant effect of Time, *F*(1, 666) = 10.847, *p* < 0.001, *η*_*p*_^*2*^ = 0.16, was qualified by a significant Employment Status × Time interaction, *F*(1, 666) = 8.034, *p* < 0.05, *η*_*p*_^*2*^ = 0.012. Post-hoc analyses revealed higher levels of depression in recently unemployed compared to employed at both waves (both *p’s* < 0.001). However, there was a significant increase of depression levels at T2 in the unemployed group, *p* < 0.001, whereas the employed group showed similar lower depression levels at both times, *p* = 0.580 (see Fig. 1). This pattern was also found in anxiety levels. There was a significant effect of Time, *F*(1, 666) = 0.590, *p* = 0.443, *η*_*p*_^*2*^ = 0.01, accounted by an Employment Status x Time significant interaction, *F*(1,666) = 16.01, *p* < 0.001, *η*_*p*_^*2*^ = 0.023. Post-hoc analyses revealed that recently unemployed had higher levels of anxiety than employed in both waves, both *p’s* < 0.001, and also showed a significant increase of anxiety levels at T2, *p* < *0.0*01. This change across time was inverse in the employed group, who decreased their anxiety levels over time *p* < *0.0*01(see Fig. 2). As for controllability appraisals and resilience levels, there were no changes from T1 to T2 (Time effects: *F*(1,666) = 2.17, *p* = 0.140, *η*_*p*_^*2*^ = 0.003, and *F*(1,666) = 0.081, *p* = 0.776, *η*_*p*_^*2*^ = 0.000, respectively). However, in both cases, Employment Status showed significant main effects (for controllability: *F*(1,666) = 32.71, *p* < 0.001, *η*_*p*_^2^ = 0.047, and for resilience: *F*(1,666) = 20.84, *p* < 0.001, *η*_*p*_^*2*^ = 0.032,), showing that recently unemployed compared to employed individuals had lower levels of perceived controllability and lower levels of resilience across both waves.

### Relationships between variables in the study

The full set of relations under study (i.e., associations between controllability, use of coping strategies and psychological symptom and resilience levels), is shown in Table 3. Controllability levels in the first wave were negatively related to depression, anxiety and positive related to resilience in both the first wave and the second wave. Controllability in wave 1 was also related to higher uses of active coping and reappraisal across the follow-up, but not to the use of distraction. Furthermore, at both waves, depression was positively related to both anxiety and the use of distraction, and negatively related to controllability and resilience. Anxiety in both waves was also positively related to the use of distraction, and negatively related to resilience. Resilience in both waves was further positively related to the use of active coping and reappraisal, and negatively related to the use of distraction. Finally, in terms of the relation between the uses of coping strategies, all these relations were positive and statistically significant.

**Table 3:** Bivariate correlations between the main study variables.

Measure *r* (*P*-value)	Controllability wave 1	Depression wave 1	Anxiety wave 1	Resilience wave 1	Depression wave 2	Anxiety wave 2	Resilience wave 2	Active Coping	Reappraisal	Distraction
Controllability wave 1	1									
Depression wave 1	− .28**	1								
Anxiety wave 1	− .26**	.81**	1							
Resilience wave 1	.54**	− .34**	− .36**	1						
Depression wave 2	− .31**	.76**	.70**	− .36**	1					
Anxiety wave 2	− .26**	.70**	.77**	− 35**	.84**	1				
Resilience wave 2	.49**	− .34**	− .36**	.77**	− .38**	− .38**	1			
Active Coping	.19**	.03	.07	.12**	− .01	.02	.13**	1		
Reappraisal	.12**	.06	.08	.08*	.05	.04	.11**	.40**	1	
Distraction	− .03	.28**	.30**	− .12*	.26**	.30**	− .15**	.33**	.28**	1

**Correlations significant at p < 0.01.

### Path models

**Figure 3:**
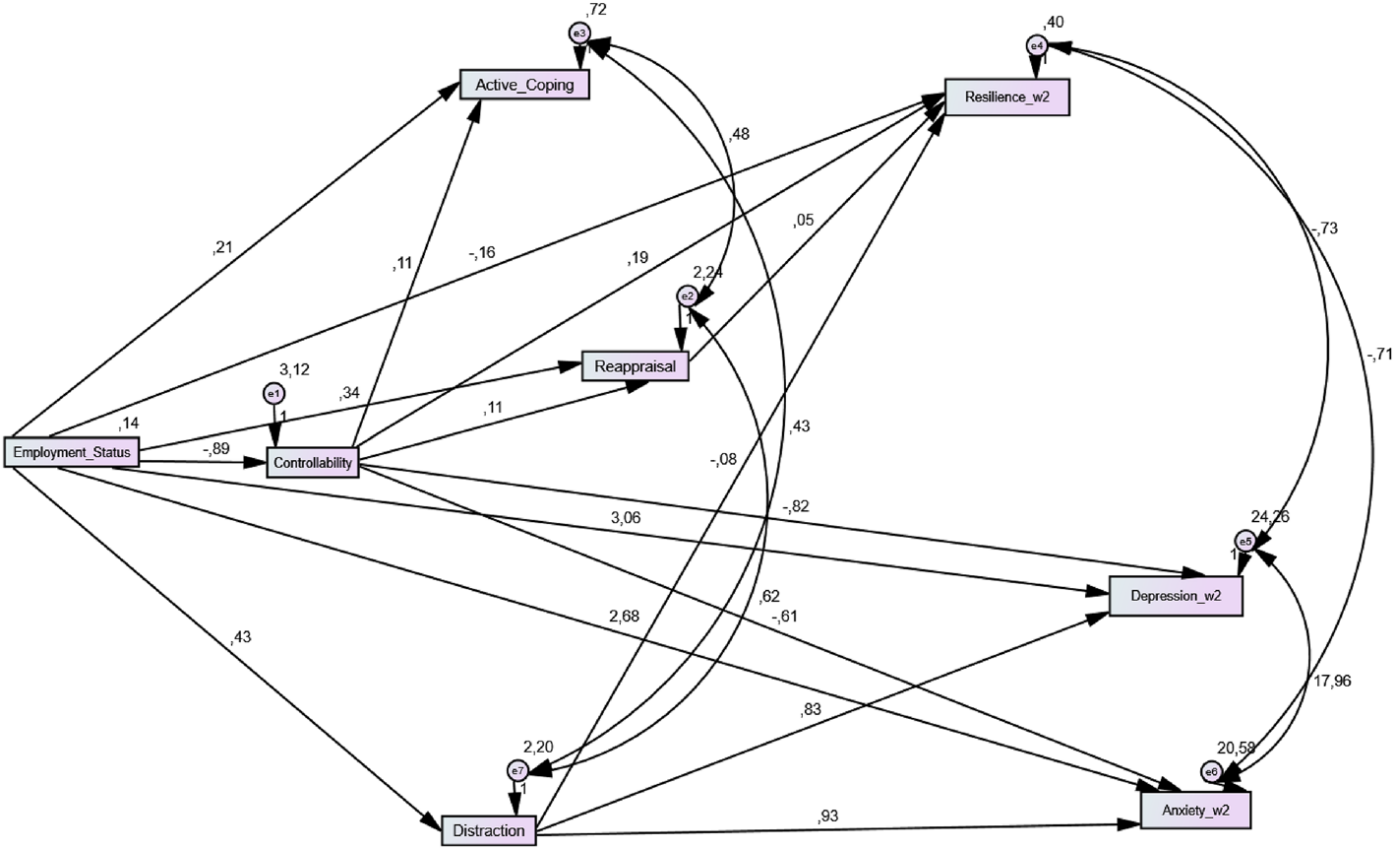
Initial path model including mediating effects of psychological mechanisms in the relationship between employment status and time 2 psychological health outcomes.

Based on the previous results regarding the relations between the hypothetical mediators and psychological outcomes, we tested the hypothesized first path model (see Fig. 3). All the goodness of fit indices were very good and are shown in Table 4.

**Table 4:** Goodness of fit indices for the main tested path model.

	*χ*^*2*^ (gl)	*P*	*χ*^*2*^/gl	CFI	TLI	RMSEA (90% CI)
Model	6.11 (6)	> .05	1.02	1.00	1.00	.01 (.01/.05)

Indirect effects in the model were then tested using a bias-corrected bootstrap estimation (2000 bootstrap samples with 95% confidence interval). As shown in Table 5, significant indirect effects were found between employment status and depression, anxiety, resilience and active coping, via controllability appraisal, all *p*’s < 0.001. Additionally, the effect of employment status on resilience was indirectly accounted via the path of controllability and reappraisal, *p* = 0.001 (i.e., employment status → controllability → reappraisal → resilience). Further, there was a significant indirect effect of employment status on resilience via reappraisal only, *p* = 0.014. Finally, analyses also supported statistically further significant indirect effects of employment status on depression, anxiety and resilience, via its relationship with the use of distraction, all *p’*s < 0.005.

**Table 5:** Bootstrap mediational analyses in the first path model considering wave 2 outcomes.

	Indirect effects (95% CI)			
	Lower	Upper	SE	*P*
**Indirect effect via controllability**				
Employment Status → Depression	.37	1.18	.72	.001
Employment Status → Anxiety	.28	.92	.54	.001
Employment Status → Resilience	− .25	− .09	− .17	.001
Employment Status → Active Coping	− .16	− .05	− .09	< .001
**Indirect effect via controllability and reappraisal**				
Employment Status → Resilience	− .01	− .01	− .01	.001
**Indirect effect via and reappraisal**				
Employment Status → Resilience	.01	.04	.02	.014
**Indirect effect via distraction**				
Employment Status → Depression	.10	.65	.35	.004
Employment Status → Anxiety	.11	.70	.39	.005
Employment Status → Resilience	− .07	− .01	− .03	.003

**Figure 4:**
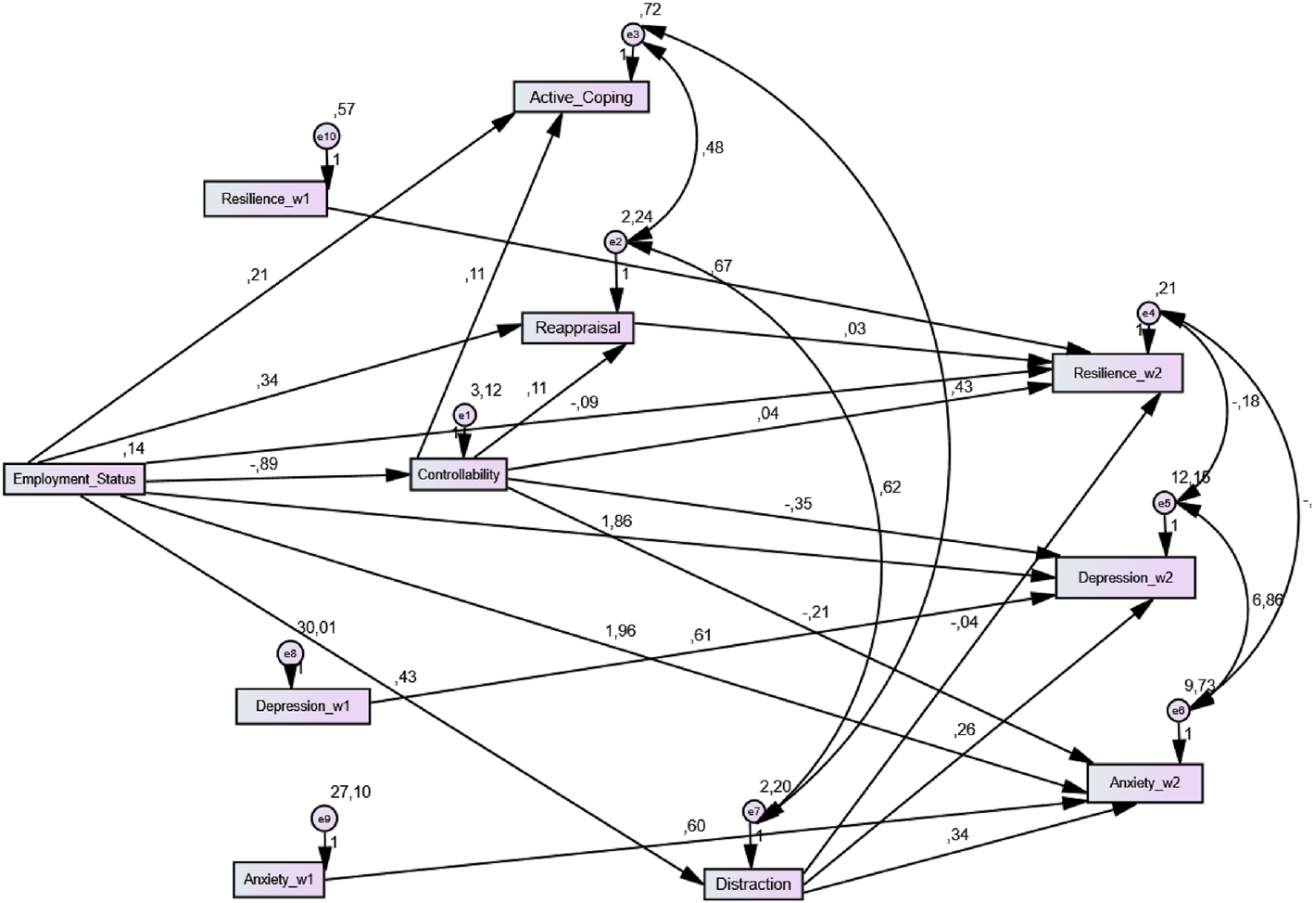
Final path model of the relation between Employment Status and changes in psychological health outcomes from Time 1 to Time 2.

The final model (i.e., modelling the change in outcome measures, via inclusion of their wave 1 predictors; see Fig. 4) confirmed that the indirect effects supported in the main path model remained all significant, supporting that the specific paths of mediation not only accounted for wave 2 individual levels but also for actual changes from wave 1 to wave 2 in depression, anxiety and resilience levels. The full set of supported indirect effects in the final path model are shown in Table 6.

**Table 6:** Bootstrap mediational analyses of the final path model considering change wave 1 to wave 2 outcomes.

	Indirect effects (95% CI)			
	Lower	Upper	SE	*P*
**Indirect effect via controllability**				
Employment Status → Depression	.14	.57	.31	.001
Employment Status → Anxiety	.06	.38	.18	.001
Employment Status → Resilience	− .07	− .01	− .04	.002
Employment Status → Active Coping	− .16	− .05	− .09	< .001
**Indirect effect via controllability and reappraisal**				
Employment Status → Resilience	− .01	− .01	− .01	.006
**Indirect effect via and reappraisal**				
Employment Status → Resilience	.01	.03	.01	.021
**Indirect effect via distraction**				
Employment Status → Depression	.02	.26	.11	.009
Employment Status → Anxiety	.04	.29	.14	.003
Employment Status → Resilience	− .04	− .01	− .02	.001

## Discussion

The present study aimed to test the hypothetical paths through which psychological mechanisms (i.e., controllability appraisals, coping strategies’ use) modulate the psychological impact of and facilitate the adaptation to a stressful situation such as job loss. These factors were monitored during the first stages of unemployment (i.e., first 3 months since job loss), a period in which the higher levels of psychological problems related to unemployment tend to occur^12,14^.

The first set of results of this study replicates previous evidence on the marked impact on the psychological functioning of job loss. Recently unemployed individuals had higher levels of depression and anxiety, and lower levels of self-reported resilience than employed individuals. These results support Hypothesis 1 and are in line with findings from previous research. Higher levels of depression and anxiety have been found in recently unemployed compared to employed people^6,17^. Furthermore, results on reduced stress resilience are also in line with previous literature finding large impacts of job loss on stress levels and general distress^15,45^. Analyses of differences between recently unemployed and employed individuals further supported Hypothesis 2. Recently unemployed individuals reported lower controllability levels after job loss compared to employed individuals, in line with previous studies^21^.

Concerning differences in these variables across time (i.e., 1 month follow-up), depression and anxiety were already higher in recently unemployed compared to employed individuals at the first assessment (i.e., immediately following job loss) but tended to further increase as the unemployment lengthened, as found in previous studies^11,12^. As for perceived resilience and controllability levels, results showed they were already lower in unemployed compared to employed individuals since the first assessment, with no differences across time.

Path analyses showed that employment status was directly related to worst psychological outcomes following job loss (i.e., higher levels of depression and anxiety, and lower levels of stress resilience), providing further support to Hypothesis 1. As for Hypothesis 3, regarding the relation of controllability appraisals and the differential use of coping strategies, results from the full model partially supported our predictions—namely, that lower controllability would predict higher use of reappraisal and distraction and that higher controllability would predict higher active coping. Specifically, a higher perception of controllability was directly related to higher use of reappraisal and active coping, but there were no significant relations between controllability and distraction. The results for active coping are in line with previous predictions^30^. However, the results for reappraisal are contrary to findings from previous research showing that higher use of reappraisal was related to lower appraisals of controllability^29,32^. This difference in our results could be at least in part explained by the pandemic situation during which the study was conducted. The COVID-19 pandemic generated an unsteady context at the time of the present study, and participants might have shown general high levels of experienced uncertainty and uncontrollability. Thus, only individuals who had more preserved appraisals of controllability in that situation might have been able to use adaptive strategies such as reappraising negative situations to reduce their psychological impact. In this regard, some experimental studies show that the specifically adaptive use of reappraisal under uncontrollable stressors would only be evidenced when those stressors are characterized by low intensity/demands^46,47^. Therefore, it is possible that at conditions of major stress, such as the ones resulting from job loss, adaptive coping strategies varied as a function of perceived controllability, in contrast to other forms of stressors with lower intensity levels. Moreover, the different time frames used to evaluate these interplays among studies might also affect results. In this sense, the result of a no significant relationship between lower controllability and higher use of distraction is contrary to the results found by authors like David and Suls^31^, who monitored these relations in the context of forms of avoidant coping during daily functioning. Overall, these findings invite to further advance on the understanding of psychological pathways of adaptation during unemployment using different types of measures (i.e., global trait-based measures, daily reports, momentary assessments) and methods (ecological assessments to monitor daily dynamics, longer longitudinal designs to fully understand these interplays across the entire unemployment scope).

As for the specific contributions of the use of each form of coping strategy to psychological outcomes during unemployment, the results partially confirm the predictions established in Hypothesis 4 (i.e., the use of reappraisal will decrease the levels of depression and anxiety, and will increase the levels of resilience, whereas the use of distraction and active coping will have the opposite consequences). Reappraisal predicted subsequent higher levels of resilience, in line with previous studies pointing out its role to promote adaptive functioning during the experience of stress (i.e.,^29^). Further, and in line with our hypothesis, distraction predicted subsequent higher levels of depression and anxiety, and lower levels of resilience, which is also in line with previous results (e.g.,^28^). The results of reappraisal and distraction showed two different pathways towards both psychological adjustment and maladjustment during the initial months of unemployment. First, a resilient pathway was associated with the use of reappraisal in the early stages of unemployment. Second, a maladaptive pathway was associated with greater use of distraction in the early stages of unemployment. In contrast, results did not support a relationship between active coping and any psychological outcome. This contrasts with previous findings (e.g.,^24,48^) showing that higher use of active coping during the initial stages of unemployment might lead to higher psychological distress, despite that it can also facilitate reemployment opportunities at the mid-term. The lack of connection between active coping and immediate psychological functioning in our study might be partly explained by the moment at which the study was conducted. As mentioned above, the study was conducted at the beginning of the COVID-19 pandemic, a moment where economic activity mostly stopped, and that was characterized by large uncertainty. This might have limited the possibilities of participants to use this sort of strategy, reducing the capacity of the study to establish the psychological consequences of its use. Nonetheless, and importantly, the model also showed that the relation of employment status with subsequent psychological outcomes can be indirectly accounted for by the interplay between controllability appraisals and coping strategies, in line with our last prediction (Hypothesis 5). This highlights the importance of considering the specific paths of influence between these factors to fully understand psychological functioning in the face of a stressful situation such as job loss^20^.

Ultimately, the evidence for separate paths of influence of recent unemployment on psychological functioning outcomes through mechanisms of controllability appraisal and coping strategy use has some potentially important practical implications. Interventions aimed to promote psychological well-being and adaptive functioning of recently unemployed people should focus both on reducing the use of distraction and improving the perception of controllability over potential problems following job loss. To tackle this, programs might include training courses, including work-related activities (e.g., create or update curriculum vitae or job interview training) to enhance coping strategies’ use. Programs might also benefit from including cognitive restructuring techniques to improve individuals’ controllability appraisals and to facilitate other forms of adaptive coping and emotion regulation during the initial stages of unemployment. Through those interventions, recently unemployed individuals might benefit from reducing associated psychological symptoms, increasing resilience to ongoing stressors and ultimately improving their probabilities for reemployment.

Despite the relevance of the findings, some limitations must be acknowledged. First, as mentioned above, conducting the study in the context of the Covid-19 pandemic represents a doubled-edged opportunity. At the time of the study (i.e., wave 1), Spain was in the middle of a very restrictive lockdown due to COVID-19 health-issues reaching peak levels and the evaluation was completed when the de-escalation process was only beginning (i.e., wave 2). In this sense, it can be assumed that the tested coping strategies were used by the unemployed participants in the study to deal with both types of stressors (i.e., health vs. economic), being difficult to separate their effects in mental health of these different forms of major stress. Thus, although COVID-19 pandemic was a crucial moment to run naturalistic psychosocial studies, the constant broader stressful circumstances occurring during the study might have partly affected the comparability of results with those from other studies on recent unemployment. To solve this issue and to facilitate generalization of our findings, future research should analyse if these results are replicated in less convulsive stages with a greater degree of social stability. Another limitation was that some of the main variables under study (controllability appraisal, coping strategies’ use) were indexed through single items. Future research should thus extend our framework and test our predictions using other forms of validated questionnaires. Beyond such limitations, the results of this study may help to inform future advanced research to fully disentangle (and promote) the mechanisms implicated in psychological adjustment to job loss and unemployment. Ultimately, these results may assist researchers, job psychologists and government institutions to identify specific pathways implicated in the development of (and protection against) psychological problems related to job loss, informing new venues to intervene them, further maximizing the probabilities of reemployment.

## Data Availability

The data that support the findings of this study are available from the corresponding author, upon reasonable request.
